# Persistent Left Superior Vena Cava and Partial Anomalous Pulmonary Venous Return in an Old Asymptomatic Female Patient

**DOI:** 10.4061/2009/152164

**Published:** 2009-11-30

**Authors:** Tayfun Sahin, Teoman Kilic, Umut Celikyurt, Ulas Bildirici, Dilek Ural

**Affiliations:** ^1^Department of Cardiology, Kocaeli University Medical Faculty, Umuttepe Yerleskesi, 41380 Kocaeli, Turkey; ^2^Department of Cardiology, Nevsehir State Hospital, 50300 Nevsehir, Turkey

## Abstract

Persistent left superior vena cava is a rare congenital venous anomaly. It results from failure of closure of the left anterior cardinal vein during cardiac development. It is usually asymptomatic but can be associated with other congenital cardiac defects including atrial septal defects, ventricular septal defects, endocardial cushion defects, tetralogy of Fallot and rhythm disturbances. PLSVC should be considered in the presence of a dilated coronary sinus on transthoracic echocardiography. The diagnosis can be made when injection of contrast in left antecubital vein results in enhancement of the dilated coronary sinus before right atrium. MRI, CT-scan and catheterisation can be used to confirm the diagnosis.

## 1. Case Report

**Figure 1:**
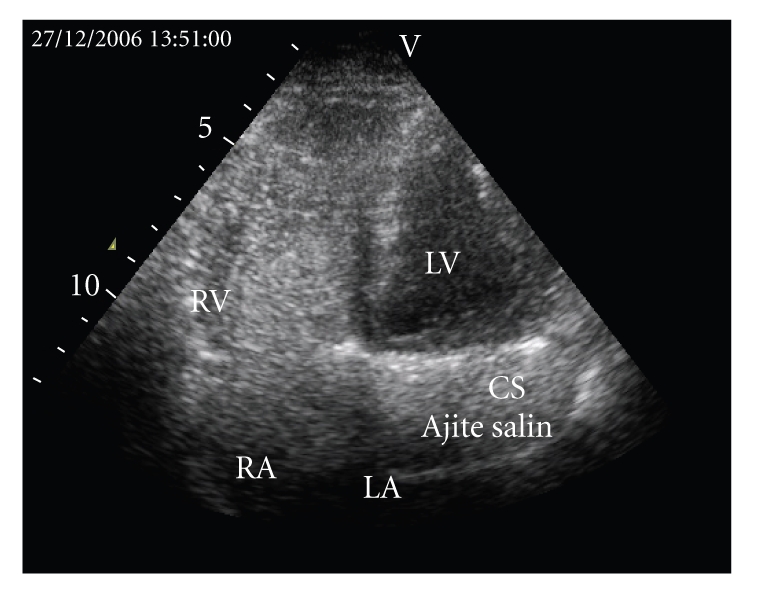
Passage of the contrast into the right atrium via coronary sinus on apical 4 chamber view of transthoracic echocardiography. LA: Left atrium, LV: Left ventricle, RV: Right ventricle, RA: Right atrium; CS: Coronary sinus.

**Figure 2:**
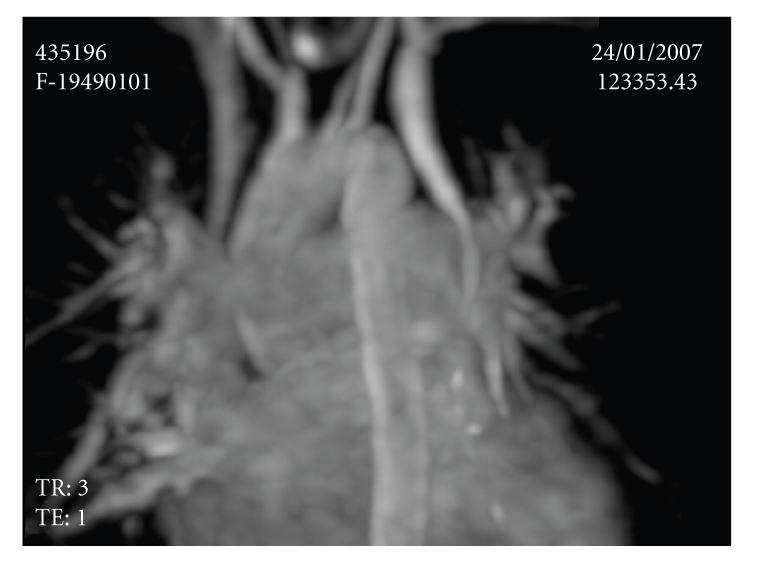
MRI showing 2 superior vena cavas, one on the right and the other on the left side.

A-57-year old asymptomatic female patient was referred to our echocardiography laboratory for etiologic evaluation of a systolodiastolic murmur over the mitral and aortic areas and 2/6 pansystolic murmur over the tricuspid area. Electrocardiography showed sinus rhythm and incomplete right bundle branch block. Cardiothoracic ratio was increased (0.55) and pulmonary conus and pulmonary vascular structures were augmented on chest X-ray. Transthoracic echocardiography (TTE) revealed biatrial and right ventricular dilatation, enlarged main pulmonary artery, and coronary sinus. She had moderate tricuspid regurgitation, pulmonary hypertension (PAP = 50 mmHg), and normal left ventricular systolic function (EF % 64). Subcostal examination showed sinus venosus type atrial septal defect. Injection of contrast in left antecubital vein confirmed passage of the contrast into the right atrium via coronary sinus and persistent left superior vena cava (PLSVC) was diagnosed (Figure 1). Pulmonary to systemic flow ratio (QP/QS) was calculated as 2.34. Transesophageal echocardiography confirmed the diagnosis. Magnetic resonans imaging (MRI) was done to interpret the vascular anatomy. MRI showed two superior vena cavas, one on the left and the other on the right side. Superior vena cava on the right side joined to the right upper pulmonary vein before draining to the right atrium and left superior vena cava drained to the right atrium via coronary sinus (Figure 2). The patient was informed about her medical condition. Although she was asymptomatic, cardiac catheterization and following surgical repair was suggested to the patient because of high pulmonary to systemic flow ratio but she did not approve any invasive procedure.

## 2. Discussion

Persistent left superior vena cava (PLSVC) is a relatively rare congenital venous anomaly occurring in approximately 0.3% to 0.5% of the normal population and 3% to 10% of patients with congenital heart disease [1, 2]. It is the most common congenital anomaly of the systemic veins [2, 3]. Failure of closure of the left anterior cardinal vein during cardiac development results in PLSVC [4]. It usually drains into the right atrium via an enlarged coronary sinus. It may also drain into the left atrium or a pulmonary vein. 

Patients with left superior vena cava (LSVC) usually have a normal right superior vena cava and the condition is infrequently detected and is hemodynamically insignificant. Other associated congenital cardiac defects (atrial septal defects, ventricular septal defects, endocardial cushion defects, and tetralogy of Fallot) and high incidence of rhythm disturbances may be detected in patients with PLSVC [5]. Wolf-Parkinson-White syndrome, sick sinus syndrome, sinus bradycardia, and sudden death have been reported in patients with PLSVC [5]. 

Chest radiography, transthoracic echocardiography, transesophageal echocardiography, magnetic resonans imaging, and computed tomography are modality of techniques used in the diagnosis of these congenital anomalies.

In conclusion, PLSVC when in isolation may be asymptomatic for many years only to become symptomatic late in life. Transthoracic echocardiography may be helpful in the diagnosis of these anomalies. PLSVC should be considered in the presence of a dilated coronary sinus on transthoracic echocardiography and the diagnosis can be easily made if the contrast is seen first in coronary sinus before arriving in the right atrium. Further investigations (MRI, CT-scan, catheterisation) are needed to confirm the diagnosis in the presence of abnormal findings on echocardiography. We have confirmed the diagnosis with MRI technique. An associated congenital anomaly should also be excluded in these patients.
